# Accuracy of Raman spectroscopy in the diagnosis of Alzheimer's disease

**DOI:** 10.3389/fpsyt.2023.1112615

**Published:** 2023-03-16

**Authors:** Yanmei Xu, Xinyu Pan, Huan Li, Qiongfang Cao, Fan Xu, Jianshu Zhang

**Affiliations:** ^1^General Practice Ward/International Medical Center Ward, General Practice Medical Center, West China Hospital, Sichuan University, Chengdu, Sichuan, China; ^2^School of Pharmacy, Chengdu Medical College, Chengdu, Sichuan, China; ^3^Department of Public Health, Chengdu Medical College, Chengdu, Sichuan, China

**Keywords:** meta-analysis, Raman spectroscopy, diagnosis, Alzheimer's disease, sensitivity and specificity

## Abstract

**Objective:**

To systematically evaluate the accuracy of Raman spectroscopy in the diagnosis of Alzheimer's disease.

**Methods:**

Databases including Web of Science, PubMed, The Cochrane Library, EMbase, CBM, CNKI, Wan Fang Data, and VIP were electronically searched for studies on Raman spectroscopy in diagnosis of Alzheimer's disease from inception to November 2022. Two reviewers independently screened the literature, extracted data, and assessed the risk of bias in the included studies. Then, meta-analysis was performed using Meta-Disc1.4 and Stata 16.0 software.

**Results:**

A total of eight studies were finally included. The pooled sensitivity of Raman spectroscopy was 0.86 [95% CI (0.80–0.91)], specificity was 0.87 [95% CI (0.79–0.92)], positive likelihood ratio was 5.50 [95% CI (3.55–8.51)], negative likelihood ratio was 0.17 [95% CI (0.09–0.34)], diagnosis odds ratio and area under the curve of SROC were 42.44 [95% CI (19.80–90.97)] and 0.931, respectively. Sensitivity analysis was carried out after each study was excluded one by one, and the results showed that pooled sensitivity and specificity had no significant change, indicating that the stability of the meta-analysis results was great.

**Conclusions:**

Our findings indicated that Raman spectroscopy had high accuracy in the diagnosis of AD, though it still did not rule out the possibility of misdiagnosis and missed diagnosis. Limited by the quantity and quality of the included studies, the above conclusions need to be verified by more high-quality studies.

## Introduction

Alzheimer's disease (AD) leads to the degeneration of brain cells. It is characterized by decline in thinking and independence in personal daily activities (1). This is a complex multifactorial disease, involving chronic neuroinflammation and neurodegeneration (2), and is related to continuous cognitive impairment and memory loss (3). In the process of treating AD patients, not only is drug therapy such as an AChE inhibitor (4) needed, but also dementia care, which is unaffordable. Obviously, it not only increases the economic burden and emotional distress on the patients themselves, but also on their families to varying degrees (5).

Early AD patients can maintain or even improve cognitive function and their ability to perform activities of daily living with non-drug therapy (such as diet and exercise) (6). Studies show that there was a significant individual difference in quality of life scores between 12 and 36 months of follow-up (7). Moreover, the evidence demonstrated that the early treatment could protect the cognitive function more effectively (6) and the early treatment could inhibit or block the underlying pathology process. Therefore, the early diagnosis of AD is crucial for early intervention.

The current clinical diagnostic criterion for AD is mainly combined with the use of AD-specific biomarkers, including identification of a β and tau deposition, glucose metabolism depression, and brain atrophy (8). At present, magnetic resonance imaging (MRI) is an imaging technology that relies on cognitive tests to confirm AD diagnosis (9). The basic neurological standard is extracellular β-amyloid protein deposition and intracellular hyperphosphorylated tau accumulation (10). It is important to distinguish the density of these specific proteins accordingly.

Dating back to 1928, Indian scientist, Sir CV Raman, discovered the “Raman effect” (11). By analyzing the frequency of scattering light, two kinds of scattering light are found: elastic scattering and inelastic scattering (12). Raman spectroscopy uses the inelastic scattering of light by matter (13), and the principle of Raman spectroscopy is used to study the interaction between radiation (including electromagnetic radiation) and matter, which is a field covering a wide range of technologies. Raman spectroscopy has become an attractive analytical tool (11) due to its versatility, rapidity of acquisition, and simplicity of analysis. Given that solid, liquid, or gas molecules have different intensities of Raman effect, Raman spectroscopy can be used to characterize the properties of substances (12). Raman spectroscopy is particularly important in early diagnosis of disease because molecular changes caused by cell differentiation, mitosis, and apoptosis can be detected through Raman spectroscopy (13).

At present, relevant studies have conducted AD diagnosis experiments using Raman spectroscopy, but the diagnostic value of Raman spectroscopy in AD is controversial. This study systematically evaluates the accuracy of Raman spectroscopy in diagnosing AD, providing a reference for clinical practice.

## Methods

### Search strategy

Two reviewers (LH and PXY) independently searched the PubMed, Embase, Cochrane library, CNKI, Wan fang, and library databases up to February 2022. Search terms are listed as follows: #1 TS = “Neurodegenerative diseases” OR “Alzheimer's disease;” #2 TS = Raman spectroscopy; #3 DT = (Clinical Trial OR Article); #4 DOP = (1971-01-01/2022-11-10); #5 #1 AND #2 AND #3AND #4.

### Inclusion and exclusion criteria

Inclusion criteria were: (1) Randomized controlled experiments; (2) The included study subjects were well-defined patients with AD; (3) The diagnostic tests included Raman spectroscopy; (4) The number of true positive (TP) cases, false negative (FN) cases, false positive (FP) cases, and the number of true negative (TN) cases could be obtained directly or calculated through the literature; (5) Age, gender, and race were not considered; (6) The literature language was not considered. Exclusion criteria were: (1) Animal studies; (2) Non-case-control trial literature; (3) Literature with incomplete or no experimental data, duplicate published literature, reviews, and abstracts; (4) Poor equilibrium between groups, different baseline, and the two groups could not be compared with the literature; (5) Re-published literature; (6) No described diagnostic tests.

### Data extraction

Two authors (LH and PXY) independently extracted the demographic data and treatment information, the third author (CQF) was involved when disagreement occurred. Baseline information extracted from eight studies contain the first author's name, year of publication, title, design type, and study subjects (number, age, male/female ratio). The primary outcomes included False negatives (FN), true negatives (TN), True positives (TP), and False positives (FP) with Raman spectroscopy.

### Quality assessment

The QUADAS-2 quality assessment tool (14) was used by two independent evaluators to assess the risk of bias in the included studies.

### Statistical analysis

Meta-Disc1.4 and Stata 16.0 software were used for meta-analysis. Spearman correlation coefficient was used to explore whether there was threshold effect, and *I*^2^ statistic was used to explore whether there was heterogeneity caused by non-threshold effect. If *I*^2^ > 50% or *p* < 0.05, the heterogeneity among included studies was significant. Random effects model was used for processing if only descriptive analysis was performed, and the sources of heterogeneity were further explored. If there was no significant heterogeneity, the fixed-effect model was used to combine sensitivity, specificity, positive likelihood ratio (PLR), negative likelihood ratio (NLR), diagnostic odds ratio (DOR), and area under the curve of SROC (AUR). All effect sizes were 95% CI, and the test level of meta-analysis was α = 0.05. Subgroup analysis was performed according to age of subjects, prediction methods, sample classification, and model classification. Stata 16.0 statistical software was used to draw Deeks' funnel plot to evaluate the publication bias of the included studies. *p* < 0.05 indicated the existence of publication bias.

## Results

### Flow chart and study quality

**Figure 1:**
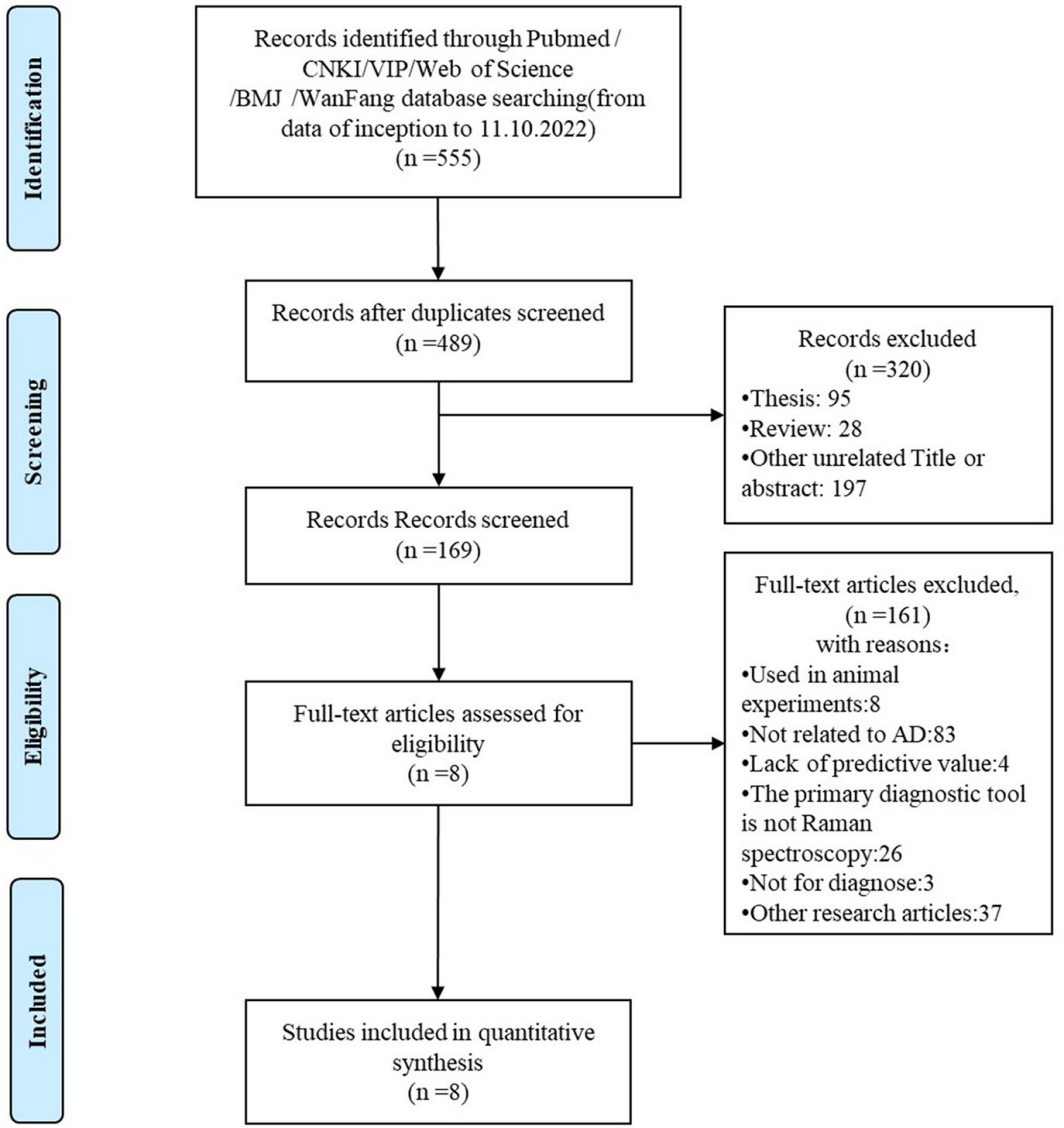
The literature screening process of the meta-analysis.

A total of 555 articles were retrieved from all databases. After 49 duplicate records were removed, among the remaining 489 relevant studies, 320 were excluded due to being reviews, meta-analyses, or case reports. The full text of the remaining 169 studies were read and 161 studies were removed after reading the full text due to incomplete data. The remaining eight papers were extracted from the corresponding data according to the data extraction requirements. The literature screening process is shown in Figure 1. The basic characteristics and inclusive and exclusive criteria of each study included are shown in Table 1 and Supplementary Table 1.

**Table 1:** Baseline characteristics of included studies.

**References**	**Country**	**Sample type**	**N (AD)**	**N (Con)**	**Age (mean ±SD)**	**Sex (male/female)**	**Cross validation**	**Diagnostic algorithm**	**Raman spectroscopy type**
Yu et al. (15)	USA	CSF	9	10	AD:79 ± 4.9; Control:76.6 ± 5.52	AD:4/5; Control:3/7	YES	CNN	CRM
Qing-Kun et al. (16)	China	Erythrocyte	16	23	AD:86 ± 3.54; Control:81.65 ± 5.8	AD:12/4; Control:17/6	NO	LDA	NA
Carlomagno et al. (17)	Italia	Blood serum	10	11	AD:73 ± 2.7; Control:65.6 ± 4.9	AD:3/7; Control:4/7	YES	LDA	CRM
Paraskevaidi et al. (18)	UK	The plasma fractions	15	15	AD:64 ± 8; Control:54 ± 18	AD:12/3; Control:6/9	YES	SVM	CRM
Carmona et al. (19)	Spain	The plasma fractions	35	12	AD:80.31; Control:73.5	AD:30/5; Control:6/6	YES	LDA	NIR
Habartová et al. (20)	Czech Rep.	The plasma fractions	35	29	AD:79 ± 8; Control:67 ± 6	NA	YES	LDA	ROA
Ryzhikova et al. (21)	USA	Blood serum	20	10	AD:74 ± 9.3; Control:68 ± 11	AD:10/10; Control:5/5	YES	MLP	NIR
Ryzhikova et al. (22)	USA	CSF	21	17	AD:72 ± 5.3; Control:71 ± 12	AD:13/8; Control:7/10	YES	ANN	NIR

CSF, cerebrospinal fluid; LDA, linear discriminant analysis; SVM, support vector machine analysis; CNN, convolutional Neural Network; MLP, multilayer Perceptron; ANN, artificial neural network; ROA, Raman optical activity spectrometer; NIR, near infrared Raman spectrometer; CRM, confocal Raman microscope.

### Quality assessment

Two reviewers independently evaluated methodological quality for each study according to the quality assessment of diagnostic accuracy studied (QUADAS) guidelines (Supplementary Table 2). All QUADAS items were used to evaluate the eligible articles. Table 2 shows the results of the evaluation of each study.

**Table 2:** Quality assessment of included studies using QUADAS questionnaire.

**Frist author**	**Q1**	**Q2**	**Q3**	**Q4**	**Q5**	**Q6**	**Q7**	**Q8**	**Q9**	**Q10**	**Q11**	**Q12**	**Q13**	**Q14**	**Score**
Xinke Yu	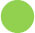	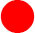	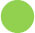	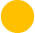	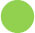	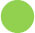	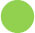	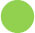	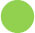	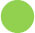	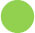	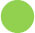	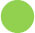	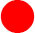	11
Luqingkun	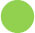	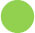	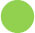	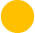	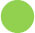	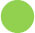	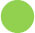	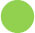	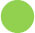	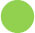	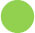	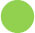	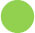	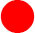	12
Carlomagno Cristiano	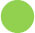	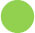	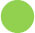	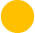	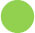	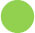	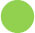	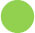	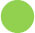	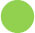	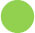	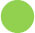	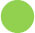	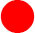	12
Maria Paraskevaidi	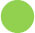	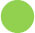	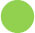	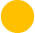	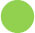	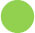	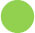	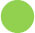	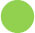	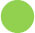	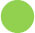	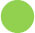	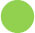	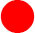	12
Pedro Carmona	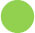	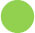	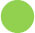	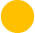	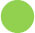	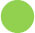	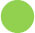	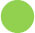	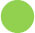	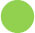	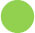	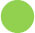	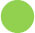	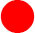	12
Lucie Habartová	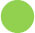	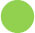	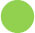	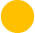	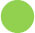	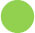	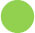	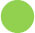	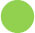	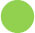	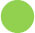	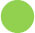	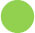	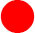	12
Elena Ryzhikova	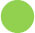	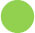	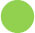	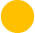	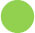	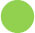	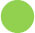	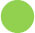	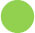	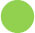	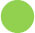	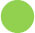	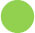	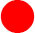	12
Elena Ryzhikova	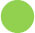	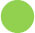	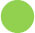	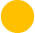	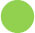	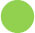	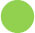	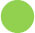	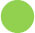	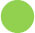	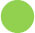	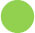	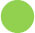	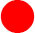	12

QUADAS, Quality assessment of diagnostic accuracy studied; 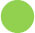, yes; 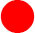, no; 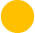, unclear.

### Analysis of diagnostic threshold

Spearman correlation analysis showed that the correlation coefficient was 0.168 *(p* = 0.691), indicating that there was no threshold effect in this study and could be combined for analysis (Supplementary Table 3).

### Combined effect size

**Figure 2:**
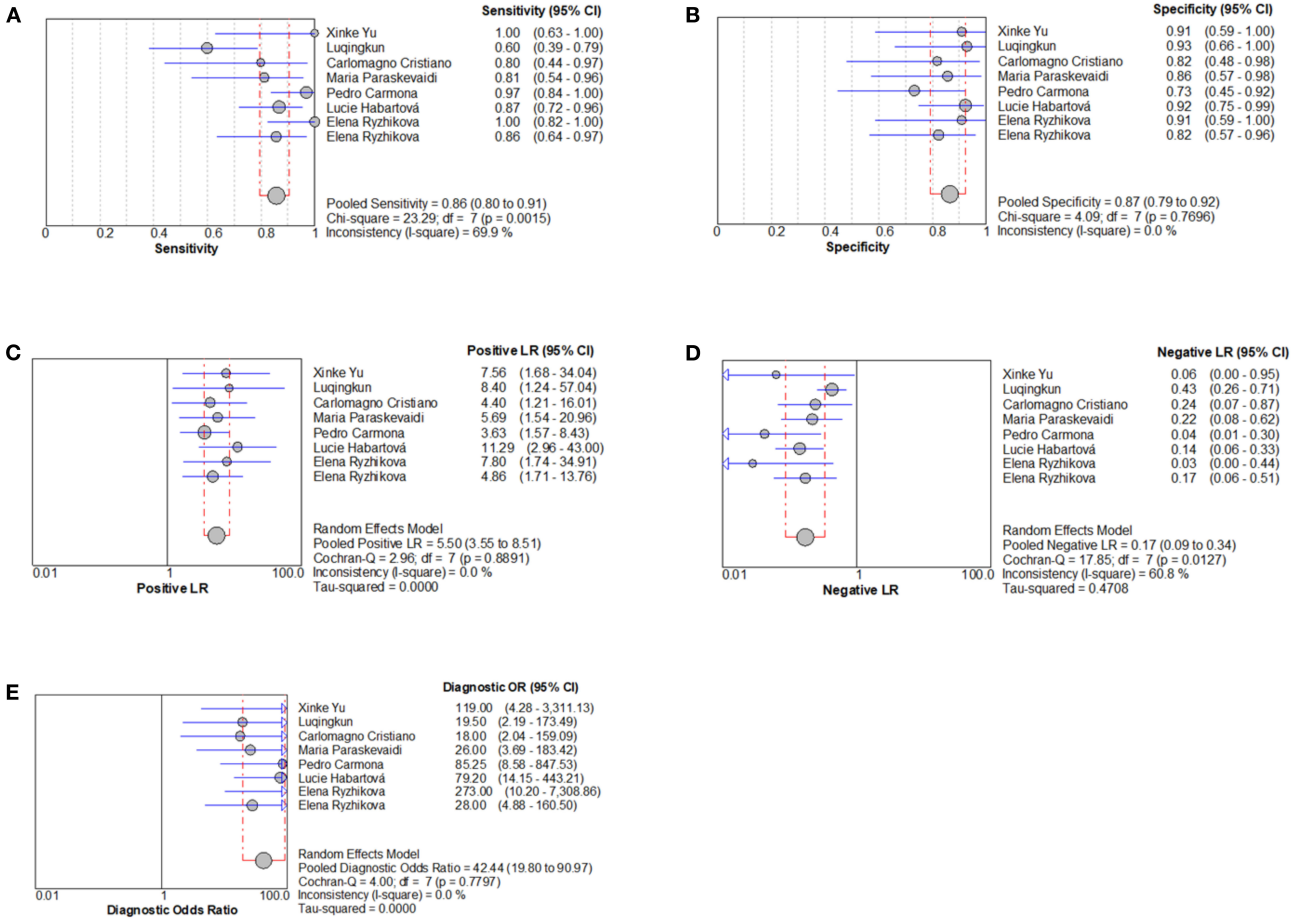
Individual study and pooled estimates of Raman spectroscopy in the diagnosis of AD. **(A)** Sensitivity, **(B)** specificity, **(C)** positive likelihood ratio, **(D)** negative likelihood ratio, and **(E)** diagnostic odds ratio.

**Figure 3:**
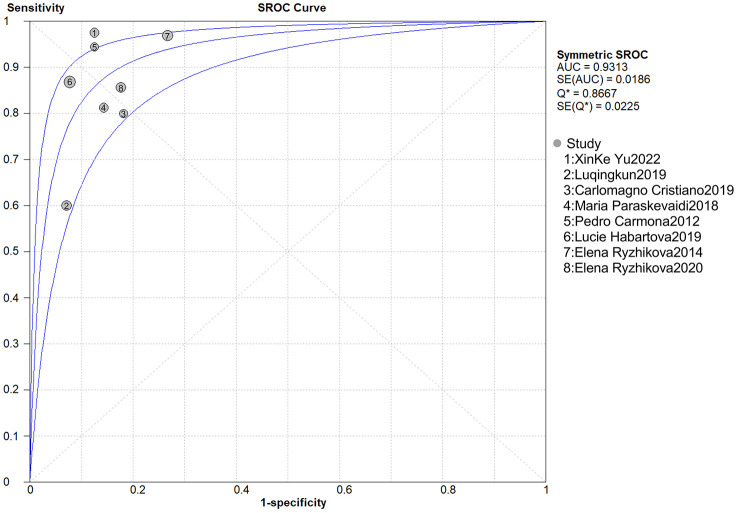
Summary receiver operating characteristics (SROC) curve of Raman spectroscopy.

The heterogeneity results of sensitivity and specificity were *I*^2^ = 69.9% *(p* = 0.002) and *I*^2^ = 0.0% *(p* = 0.770), respectively, indicating that there were some heterogeneities among included studies (Figures 2A, B). The results of random effects model meta-analysis showed that the pooled sensitivity of Raman spectroscopy was 0.86 [95% CI (0.80–0.91)], specificity was 0.87 [95% CI (0.79–0.92)], PLR was 5.50 [95% CI (3.55–8.51)], NLR was 0.17 [95% CI 0.09–0.34)], and diagnosis odds ratio (DOR) and area under the curve of SROC (AUC) were 42.44 [95% CI (19.80–90.97)] and 0.931, respectively (Figures 2A–E, 3).

### Subgroup analysis

The results of subgroup analysis showed that subject age, sample type, diagnostic algorithm, and combined other spectra were all possible sources of heterogeneity. The random effects model was used to combine the effect size. The results showed that the pooled sensitivity of cerebrospinal fluid (CSF) was higher than that of blood, but the pooled specificity was lower than that of blood. When the diagnostic algorithm was analyzed, the sensitivity and specificity of other pooled were higher than that of LDA (linear discriminant analysis), but the AUC was lower. At the age of analysis, the pooled sensitivity of <75 years old was higher than that of >75 years old, but the pooled specificity was lower. When combining other spectra, the pooled sensitivity of yes was higher than that of no, but the pooled specificity was lower (Table 3).

**Table 3:** Summary of subgroup analysis results.

**Subgroup**		**Included Studies**	**Pooled sensitivity (95% CI)**	***I^2^* (pooled sensitivity)**	**Pooled specificity (95% CI)**	***I^2^* (pooled specificity)**	**Pooled PLR (95% CI)**	**Pooled NLR (95% CI)**	**Pooled DOR (95% CI)**	**AUC**
Sample type	CSF	2	0.90 (0.73–0.98)	51.60%	0.86 (0.67–0.96)	0.00%	5.60 (2.38–13.20)	0.15 (0.06–0.41)	38.28 (8.16–179.61)	–
	Blood	6	0.85 (0.78–0.90)	75.90%	0.87 (0.78–0.93)	0.00%	5.46 (3.29–9.08)	0.18 (0.08–0.40)	43.87 (18.26–105.41)	0.935
Diagnostic Algorithm	LDA	4	0.83 (0.74–0.90)	78.70%	0.86 (0.76–0.94)	12.80%	5.12 (2.83–9.27)	0.19 (0.07–0.52)	42.40 (15.17–118.51)	0.934
	Other	4	0.91 (0.81–0.96)	58.10%	0.87 (0.75–0.95)	0.00%	5.98 (3.13–11.41)	0.16 (0.08–0.32)	42.48 (13.62–132.47)	0.912
Age	< 75	4	0.88 (0.78–0.95)	50.60%	0.85 (0.72–0.93)	0.00%	5.34 (2.86–9.99)	0.19 (0.10–0.35)	31.26 (10.85–90.05)	0.905
	>75	4	0.84 (0.76–0.91)	82.20%	0.88 (0.78–0.95)	10.30%	5.65 (3.07–10.40)	0.15 (0.04–0.52)	59.05 (19.66–177.40)	0.947
Combined other spectra	Yes	2	0.91 (0.82–0.97)	59.30%	0.85 (0.71–0.94)	62.10%	5.73 (1.81–18.10)	0.11 (0.04–0.30)	81.33 (20.51–322.55)	–
	No	6	0.82 (0.73–0.89)	71.50%	0.87 (0.78–0.94)	0.00%	5.82 (3.34–10.11)	0.21 (0.10–0.45)	31.84 (12.75–79.55)	0.922

CSF, cerebrospinal fluid; LDA, linear discriminant analysis.

### Publication bias

**Figure 4:**
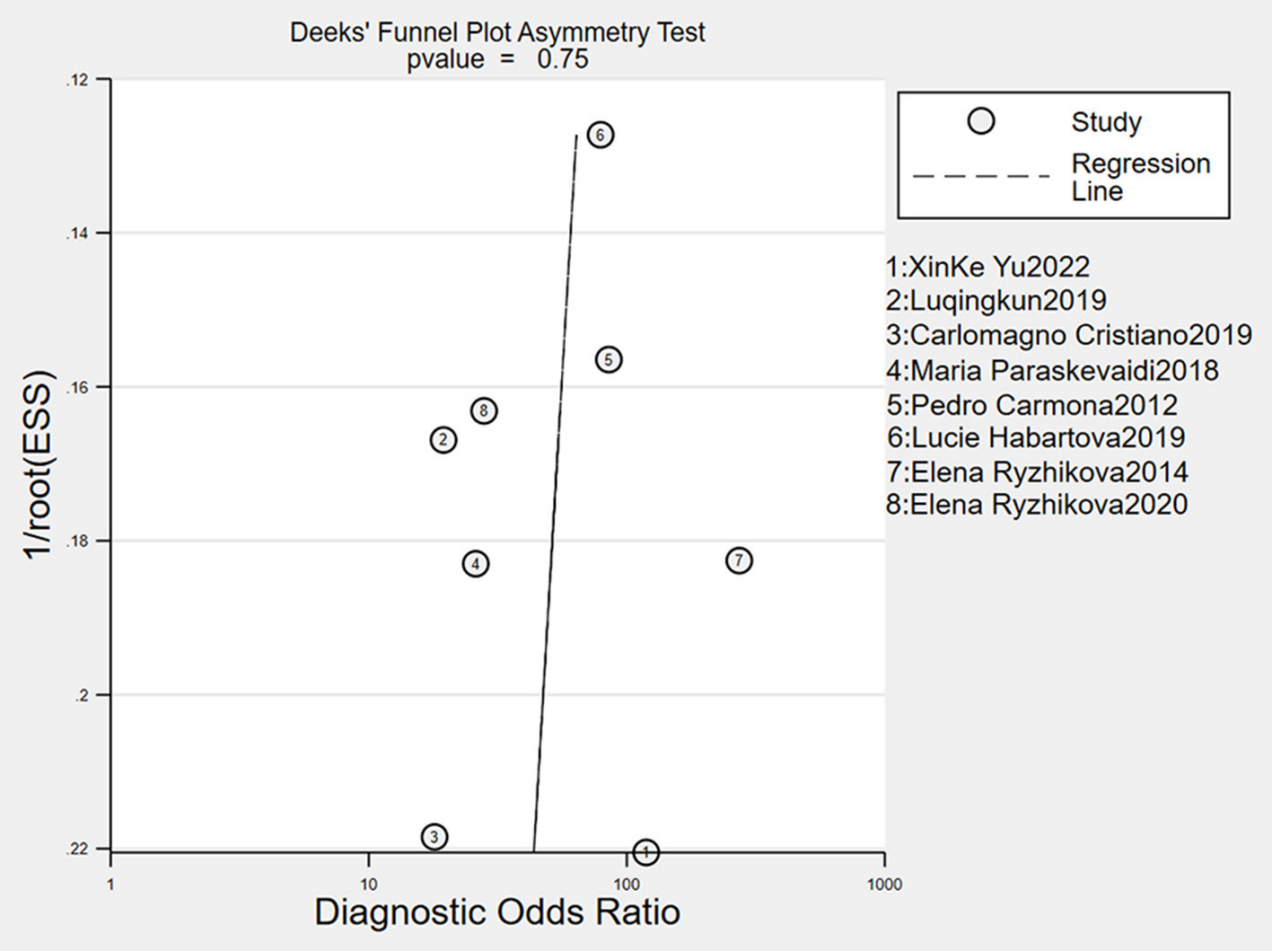
Deeks' funnel plots indicating no publication bias.

The Deeks' funnel plot asymmetry tests demonstrated that no significant publication bias was found. The funnel plots were shown in Figure 4.

### Sensitivity analysis

When each study was eliminated one by one, the combined effect amount of other studies was within 95% CI of the total effect amount, indicating that the results of this meta-analysis were relatively stable, and there was no single heterogeneity study (Table 4).

**Table 4:** The sensitivity analysis results.

**Study omitted**	**Estimate**	**95% CI**	
Yu et al. (15)	4.37	2.47	7.76
Qing-Kun et al. (16)	5.56	3.48	8.87
Carlomagno et al. (17)	4.92	2.50	9.69
Paraskevaidi et al. (18)	5.01	2.47	10.18
Carmona et al. (19)	4.34	2.42	7.79
Habartová et al. (20)	4.79	2.33	9.87
Ryzhikova et al. (21)	4.30	2.48	7.47
Ryzhikova et al. (22)	4.89	2.42	9.86
Combined	4.70	2.62	8.44

## Discussion

The application of Raman spectroscopy to clinically relevant biological species, disease pathogenesis, and diagnosis has increased rapidly over the past decade (23). The results of this study reflected the application of Raman spectroscopy in the diagnosis of neurological disease AD. It disclosed that the sensitivity and specificity of Raman spectroscopy in the diagnosis of AD were 86 and 87%. The value of PLP was 5.5 > 1, and that of NLP was 0.17 < 1, indicating that Raman spectroscopy has a high possibility of diagnosing true positive and true negative AD. The AUC is 0.93, indicating that its diagnostic efficiency was high, but there was still the possibility of missing diagnosis and misdiagnosis. Therefore, Raman spectroscopy can be used as an auxiliary diagnostic method for AD detection. Raman spectrum also showed good accuracy in the diagnosis of other diseases, such as HBV diagnosis accuracy of 93.1% (24) and cervical tissue identification overall diagnosis accuracy of 85.7% (25). Amyotrophic lateral sclerosis (ALS) based on tears and Raman spectroscopy also have good diagnostic value (26), Huefner et al. (27) also proposed Serum Raman spectroscopy as a diagnostic tool in patients with Huntington's disease. In addition, we found that Raman spectroscopy also has a good application in AD rat experiments. Such as the retina biochemical characterization of AD mouse was detected by Raman spectroscopy, with a sensitivity of 86.2%, spectra of AD-diseased mice were correctly recognized (28), and in distinguishing amyloid β-Protein in a mouse model of AD, the peak intensity ratio of Raman spectrum was different (29). Based on Raman spectrum Ab40 and Ab42 can be easily distinguished, these two peptide isomers are associated with classical vascular AD (Ab40) and parenchymal (Ab42) plaques in AD, respectively (30). And provides a well direction for the future application of AD disease. This indicates that Raman spectroscopy has a good application in the diagnosis of diseases, which is not only fast and efficient (31), but also widely used (32). It is also a non-invasive diagnostic tool (33). The high-precision detection of Raman spectroscopy is expected to reduce or replace other AD diagnostic tests.

Subgroup results showed that different sample types had different sensitivities, and CSF was more sensitive than blood samples. However, due to insufficient literature included, AUC value of CSF could not be calculated, so more studies were needed to explore the diagnostic value of Raman spectroscopy of CSF. Previously, the detection of CSF biomarkers was one of the diagnostic criteria for AD (34). This study demonstrated that Raman spectroscopy could also be used as another means to observe CSF. Besides, by analyzing blood samples, which were also subdivided into serum, plasma, and erythrocytes, pooled AUC showed better diagnostic efficacy (0.935). In addition, we noticed that the AUC of Raman spectrum in the diagnosis of AD patients older than 75 years was 0.947, indicating higher diagnostic efficiency. Many studies have also proven that in different levels of AD, plasma NFL (neurofilament light) was particularly high in patients with MCI (mild cognitive impairment) and patients with AD dementia with Amyloid-b pathology (35), which indicates that Raman spectroscopy can be applied to the diagnosis stage of AD disease. When analyzing whether to combine other spectra for diagnosis, we found that the sensitivity of AD diagnosis combined with other spectra was higher. However, due to insufficient literature included, the AUC value could not be calculated, which is also worth further exploration. In the application of different diagnostic models, we found that there were differences in the sensitivity and specificity of different models, this study found that there are diagnostic models based on CNN (15, 30), LDA (16, 17, 19, 20), SVM (18), MLP (21) and ANN (22), and the AUC value of LDA used in more studies was 0.934, which was not much different from that of pooled AUC, which seemed to suggest that LDA was suitable for the diagnosis and recognition of Raman spectroscopy in AD. Due to the limited number of other types of diagnostic models, it is not possible to calculate the AUC of other types of diagnostic models. However, it can be found from the data analysis of a single paper that ANN has the highest sensitivity and CNN has the highest specificity. The diagnostic algorithm on AD mouse is also different, for example, by contacting monolayer graphene with the brain slices, the accuracy was increased from 77 to 98% in machine learning classification (36). In other studies based on Raman spectroscopy, machine learning metrics and random forest algorithm predicted capillary-lining cell identities with 90% accuracy (37). Different diagnostic algorithms differ in the application of Raman spectrum, and we expect more and richer studies to prove it.

## Study limitation

This study also has some limitations. There were few studies included in this study, which was insufficient to reflect the biodiversity of AD patients. Although there was no publication bias in our meta-analysis, it is worth noting that any meta-analysis cannot eliminate bias, so more research on the application of Raman spectroscopy in AD diagnosis is needed.

## Conclusion

Our research showed that Raman spectroscopy was an effective and accurate tool for diagnosing AD, though it still could not rule out the possibility of missed diagnosis and misdiagnosis. The number of studies included was limited. More high-quality studies are needed to verify the above conclusions.

## Data availability statement

The original contributions presented in the study are included in the article/Supplementary material, further inquiries can be directed to the corresponding authors.

## Author contributions

YX and JZ developed the study concept and drafted the manuscript. HL and XP screened the reference and extracted the data from the literature. QC accessed the basic information from the study. YX and JZ analyzed and interpreted the data. All authors contributed to the article and approved the submitted version.

## References

[1] Breijyeh ZA-O Karaman RA-O. Comprehensive review on alzheimer's disease: causes and treatment. Molecules. (2020) 25:5789. 10.3390/molecules25245789 33302541 PMC7764106

[2] Goyal D Ali SA Singh RK. Emerging role of gut microbiota in modulation of neuroinflammation and neurodegeneration with emphasis on Alzheimer's disease. Prog Neuropsychopharmacol Biol Psychiatry. (2021) 106:110112. 10.1016/j.pnpbp.2020.110112 (1878-4216 ). 32949638

[3] Reiss AA-O Glass AD Wisniewski T Wolozin B Gomolin IH Pinkhasov A . Alzheimer's disease: many failed trials, so where do we go from here? J Investig Med. (2020) 68:1135–40. 10.1136/jim-2020-001297 32699179 PMC7872435

[4] Ferreira-Vieira TH Guimaraes IM Silva FR Ribeiro FM. Alzheimer's disease: targeting the cholinergic system. Curr Neuropharmacol. (2016) 14:101–15. 10.2174/1570159x13666150716165726 26813123 PMC4787279

[5] Alzheimer's disease facts and figures. Alzheimers Dement. (2021) 17:327–406. 10.1002/alz.12328 33756057

[6] Trigg R Jones RW Knapp M King D Lacey LA. The relationship between changes in quality of life outcomes and progression of alzheimer's disease: results from the dependence in ad in England 2 longitudinal study. Int J Geriatr Psychiatry. (2015) 30:400–8. 10.1002/gps.4150 24920081

[7] Vogel A Bhattacharya S Waldorff FB Waldemar G. Proxy-rated quality of life in Alzheimer's disease: a three-year longitudinal study. Int Psychogeriatr. (2012) 24:82–9. 10.1017/S1041610211001128 21729415

[8] Graff-Radford J Yong KXX Apostolova LG Bouwman FH Carrillo M Dickerson BC . New insights into atypical Alzheimer's disease in the era of biomarkers. Lancet Neurol. (2021) 20:222–34. 10.1016/S1474-4422(20)30440-3 33609479 PMC8056394

[9] Lombardi G Crescioli G Cavedo E Lucenteforte E Casazza G Bellatorre AG . Structural magnetic resonance imaging for the early diagnosis of dementia due to Alzheimer's disease in people with mild cognitive impairment. Cochrane Database Syst Rev. (2020) 3:CD009628. 10.1002/14651858.CD009628 32119112 PMC7059964

[10] Zhang Y Geng R Tu Q. Gut Microbial Involvement in Alzheimer's Disease Pathogenesis. Aging. (2021) 13:13359–71. 10.18632/aging.202994 33971619 PMC8148443

[11] Gala U Chauhan H. Principles and applications of raman spectroscopy in pharmaceutical drug discovery and development. Expert Opin Drug Discov. (2015) 10:187–206. 10.1517/17460441.2015.981522 25399993

[12] Wang WT Zhang H Yuan Y Guo Y He S-X. Research progress of raman spectroscopy in drug analysis. AAPS PharmSciTech. (2018) 19:2921–8. 10.1208/s12249-018-1135-8 30091063

[13] Ember KJI Hoeve MA McAughtrie SL Bergholt MS Dwyer BJ Stevens MM . Raman spectroscopy and regenerative medicine: a review. Nature. (2017) 2:12. 10.1038/s41536-017-0014-3 29302348 PMC5665621

[14] Whiting PF Rutjes AW Westwood ME Mallett S Deeks JJ Reitsma JB . Quadas-2: a revised tool for the quality assessment of diagnostic accuracy studies. Ann Intern Med. (2011) 155:529–36. 10.7326/0003-4819-155-8-201110180-00009 22007046

[15] Yu X Srivastava S Huang SA Hayden EY Teplow DB Xie Y-H. The feasibility of early Alzheimer's disease diagnosis using a neural network hybrid platform. Biosensors. (2022) 12:753. 10.3390/bios12090753 36140138 PMC9496690

[16] Qing-Kun L Gao-Zhong H Jie C Mei-Zhen H Dong-Mei D Qian Z. Diagnositc value of surface-enhanced Raman spectroscopy of peripheral erythrocytes for geriatric cognitive disorder. Chin J Mult Organ Dis Elderly. (2019) 18:300–4.

[17] Carlomagno C Cabinio M Picciolini S Gualerzi A Baglio F Bedoni MA-O. SERS-based biosensor for Alzheimer disease evaluation through the fast analysis of human serum. J Biophotonics. (2020) 13:e201960033. 10.1002/jbio.201960033 31868266

[18] Paraskevaidi MA-O Morais CLM Halliwell DE Mann DMA Allsop D Martin-Hirsch PL . Raman spectroscopy to diagnose Alzheimer's disease and dementia with lewy bodies in blood. ACS Chem Neurosci. (2018) 9:2786–94. 10.1021/acschemneuro.8b00198 29865787

[19] Carmona P Molina M Calero M Bermejo-Pareja F Martínez-Martín P Toledano A. Discrimination analysis of blood plasma associated with Alzheimer's disease using vibrational spectroscopy. J Alzheimers Dis. (2013) 34:911–20. 10.3233/JAD-122041 23302656

[20] Habartová L Hrubešová K Syslová K Vondroušová J Fišar Z Jirák R . Blood-based molecular signature of Alzheimer's disease via spectroscopy and metabolomics. Clin Biochem. (2019) 72:58–63. 10.1016/j.clinbiochem.2019.04.004 30954438

[21] Ryzhikova E Kazakov O Halamkova L Celmins D Malone P Molho E . Raman spectroscopy of blood serum for Alzheimer's disease diagnostics: specificity relative to other types of dementia. J Biophotonics. (2015) 8:584–96. 10.1002/jbio.201400060 25256347 PMC4575592

[22] Ryzhikova E Ralbovsky NM Sikirzhytski V Kazakov O Halamkova L Quinn J . Raman spectroscopy and machine learning for biomedical applications: Alzheimer's disease diagnosis based on the analysis of cerebrospinal fluid. Spectrochim Acta A Mol Biomol Spectrosc. (2021) 248:119188. 10.1016/j.saa.2020.119188 33268033

[23] Devitt G Howard K Mudher A Mahajan S. Raman spectroscopy: an emerging tool in neurodegenerative disease research and diagnosis. ACS Chem Neurosci. (2018) 9:404–20. 10.1021/acschemneuro.7b00413 29308873

[24] Tong D Chen C Zhang J Lv G Zheng X Zhang Z . Application of Raman spectroscopy in the detection of hepatitis B virus infection. Photodiagnosis Photodyn Ther. (2019) 28:248–52. 10.1016/j.pdpdt.2019.08.006 31425766

[25] Wang J Zheng CX Ma CL Zheng X-X Lv X-Y Lv G-D . Raman spectroscopic study of cervical precancerous lesions and cervical cancer. Lasers Med Sci. (2021) 36:1855–64. 10.1007/s10103-020-03218-5 33404885 PMC8594213

[26] Ami D Duse A Mereghetti P Cozza F Ambrosio F Ponzini E . Tear-based vibrational spectroscopy applied to amyotrophic lateral sclerosis. Anal Chem. (2021) 93:16995–7002. 10.1021/acs.analchem.1c02546 34905686 PMC8717331

[27] Huefner A Kuan WL Mason SL Mahajan S Barker RA. Serum Raman spectroscopy as a diagnostic tool in patients with Huntington's disease. Chem Sci. (2020) 11:525–33. 10.1039/c9sc03711j 32190272 PMC7067270

[28] Stiebing C Jahn I Schmitt M Keijzer N Kleemann R Kiliaan AJ . Biochemical characterization of mouse retina of an Alzheimer's disease model by raman spectroscopy. Chem Neurosci. (2020) 11:3301–8. 10.1021/acschemneuro.0c00420 32991138 PMC7581290

[29] Li S Luo Z Zhang R Xu H Zhou T Liu L . Distinguishing amyloid β-protein in a mouse model of Alzheimer's disease by label-free vibrational imaging. Biosensors. (2021) 11:365. 10.3390/bios11100365 34677321 PMC8533730

[30] Yu X Hayden EY Xia M Liang O Cheah L Teplow DB . Surface enhanced raman spectroscopy distinguishes amyloid β-protein isoforms and conformational states. Protein Sci. (2018) 27:1427–38. 10.1002/pro.3434 29700868 PMC6153385

[31] Akçan R Yildirim MS Ilhan H Güven B Tamer U Saglam N. Surface enhanced raman spectroscopy as a novel tool for rapid quantification of heroin and metabolites in saliva. Turk J Med Sci. (2020) 50:1470–9. 10.3906/sag-1912-196 32178510 PMC7491273

[32] Eberhardt K Stiebing C Matthäus C Schmitt M Popp J. Advantages and limitations of Raman spectroscopy for molecular diagnostics: an update. Expert Rev Mol Diagn. (2015) 15:773–87. 10.1586/14737159.2015.1036744 25872466

[33] Blanco-Formoso M Alvarez-Puebla RA. Cancer diagnosis through sers and other related techniques. Int J Mol Sci. (2020) 21:2253. 10.3390/ijms21062253 32214017 PMC7139671

[34] Blennow K Zetterberg H. Biomarkers for Alzheimer's disease: current status and prospects for the future. J Intern Med. (2018) 284:643–63. 10.1111/joim.12816 30051512

[35] Mattsson N Andreasson U Zetterberg H Blennow K. Association of plasma neurofilament light with neurodegeneration in patients with Alzheimer disease. JAMA Neurol. (2017) 74:557–66. 10.1001/jamaneurol.2016.6117 28346578 PMC5822204

[36] Wang Z Ye J Zhang K Ding L Granzier-Nakajima T Ranasinghe JC . Rapid biomarker screening of Alzheimer's disease by interpretable machine learning and graphene-assisted raman spectroscopy. ACS Nano. (2022) 16:6426–36. 10.1021/acsnano.2c00538 35333038

[37] Francis AT Manifold B Carlson EC Hu R Hill AH Men S . In vivo simultaneous nonlinear absorption raman and fluorescence (snarf) imaging of mouse brain cortical structures. Commun Biol. (2022) 5:222. 10.1038/s42003-022-03166-6 35273325 PMC8913696

