# Supplementary material for: Accuracy of Raman spectroscopy in the diagnosis of Alzheimer's disease
Source: Front Psychiatry. 2023 Mar 16;14:1112615. doi: 10.3389/fpsyt.2023.1112615 (PMC10060832; doi:10.3389/fpsyt.2023.1112615)
Supplement: Supplementary file 1 [file Table_1.docx]

| **Supplementary table 1** Baseline characteristics of included studies | | | | |
| --- | --- | --- | --- | --- |
| **First author** | **Year** | **Golden criteria of diagnosis** | **Inclusive criteria** | **Exclusive criteria** |
| Xinke Yu^[15]^ | 2022 | ADCR | NA | NA |
| Luqingkun^[16]^ | 2019 | DSM-V | Conform to diagnostic and statistical manual of mental disorders,5th edition | Any other nervous system disease that causes dementia, including Parkinson's disease, epilepsy, brain trauma, etc; Depression or other mental diseases conforming to DSM-V in the past 2 years; Have a history of alcohol, drug abuse or dependence in the past 2 years; Any blood system disease; Any significant systemic disease or unstable medical condition, including systemic lupus erythematosus, rheumatoid arthritis, acute heart failure, neurosyphilis, etc; Used drugs that affect brain function in the first 2 weeks |
| Carlomagno Cristiano^[17]^ | 2019 | ATN/CDR | 1)probable AD according to ATN criteria 3,4 with an amyloid-PET scanning session (18F-Florbetapir), in order to test for Aβ burden;  2) mild stage of the disease with a Clinical Dementia Rating (CDR) scale score between 0.5-1 20; 3) absence of MRI contraindications or brain abnormalities | NA |
| Maria Paraskevaidi^[18]^ | 2018 | NA. | NA | NA |
| Pedro Carmona^[19]^ | 2012 | DMS/NINCDS-ARDA | NA | NA |
| Lucie Habartová^[20]^ | 2019 | NA | NA | NA |
| Elena Ryzhikova^[21]^ | 2014 | NINDSADRDA | NA | NA |
| Elena Ryzhikova^[22]^ | 2020 | NINDSADRDA | NA | NA |

ADRC alzheimer’s disease research center; DSM-V diagnostic and statistical manual of mental disorders,5th edition; ATN an unbiased descriptive classification scheme for Alzheimer disease biomarkers; CDR clinical dementia rating; NA not available from original study paper or supplementary or registration information
