# Supplementary material for: Accuracy of Raman spectroscopy in the diagnosis of Alzheimer's disease
Source: Front Psychiatry. 2023 Mar 16;14:1112615. doi: 10.3389/fpsyt.2023.1112615 (PMC10060832; doi:10.3389/fpsyt.2023.1112615)
Supplement: Supplementary file 2 [file Table_2.docx]

| **Supplementary table 2** Analysis of Diagnostic Threshold | | | | | | |
| --- | --- | --- | --- | --- | --- | --- |
| Var | Coeff. | Std. Error | T | p-value | Spearman correlation coefficient | p |
| a | 3.744 | 0.389 | 9.623 | 0.0001 | 0.168 | 0.691 |
| b (1) | 0.366 | 0.316 | 1.158 | 0.2909 |  |  |
