# Supplementary material for: Accuracy of Raman spectroscopy in the diagnosis of Alzheimer's disease
Source: Front Psychiatry. 2023 Mar 16;14:1112615. doi: 10.3389/fpsyt.2023.1112615 (PMC10060832; doi:10.3389/fpsyt.2023.1112615)
Supplement: Supplementary file 3 [file Table_3.docx]

| **Supplementary Table 3** Quality assessment of diagnostic accuracy studied(QUADAS) | |
| --- | --- |
| **NO.** | **Question** |
| Q1. | Was the spectrum of patients representative of the patients who will receive the test in practice? |
| Q2. | Were selection criteria clearly described? |
| Q3. | Is the reference standard likely to correctly classify the target condition? |
| Q4. | Is the time period between reference standard and index test short enough to be reasonable? |
| Q5. | Did the whole sample, or a random selection of the sample, receive verification using a reference standard of diagnosis? |
| Q6. | Did patients receive the same reference standard regardless of the index test result? |
| Q7. | Was the reference standard independent of the index test (i.e. the index test did not form part of the reference standard)? |
| Q8. | Was the execution of the index test described in sufficient detail to permit replication of the test? |
| Q9. | Was the execution of the reference standard described in sufficient detail to permit its replication? |
| Q10. | Were the index test results interpreted without knowledge of the results of the reference test? |
| Q11. | Were the reference standard results interpreted without knowledge of the results of the index test? |
| Q12. | Were the same clinical data available when test results were interpreted as would be available when the test is used in practice? |
| Q13. | Were interpretable/intermediate test results reported? |
| Q14. | Were withdrawals from the study explained? |
